# Factors associated with community health workers’ confidence in addiction service provision in Vietnam: a cross-sectional study

**DOI:** 10.1186/s12913-026-15401-5

**Published:** 2026-08-18

**Authors:** Van Thi Nguyen, Li Li, Tuan Anh Nguyen, Ha Thi Thanh Nguyen, Than Dang Phan, Thang Hong Pham

**Affiliations:** 1 National Institute of Hygiene and Epidemiology, 1 Yersin Street, Hanoi, 100000 Vietnam; 2 Department of Psychiatry and Biobehavioral Sciences, David Geffen School of Medicine, University of California, Los Angeles, 760 Westwood Plaza, Los Angeles, CA 90024 USA

**Keywords:** Community health worker, Confidence in service provision, Job satisfaction, Vietnam

## Abstract

**Background:**

Community health workers play a critical role in expanding access to addiction services in Vietnam, particularly in the context of recent health system reforms that strengthen community-level healthcare delivery. To optimize the effectiveness of service delivery for people who use drugs in community settings, it is essential to understand factors that shape community health workers’ confidence in providing addiction-related services. This study examined individual and work-related correlates of their confidence in service provision.

**Methods:**

We utilized baseline data from an intervention trial conducted in three provinces of Vietnam. A total of 174 community health workers from 58 community health centers participated in the study. Multivariable linear regression models were employed to examine associations between confidence in service provision and key factors, including job satisfaction, training related to addiction services, attitudes toward methadone maintenance treatment, and demographic characteristics.

**Results:**

Higher confidence in addiction service provision was significantly associated with older age (β = 0.31, *p* < 0.01) and prior addiction-related training (β = 0.18, *p* < 0.05). More positive attitudes toward methadone maintenance treatment were associated with greater confidence in bivariate and partially adjusted models (β = 0.18, *p* < 0.05), but this relationship was attenuated after accounting for job satisfaction. Job satisfaction remained positively associated with confidence in the final adjusted model (β = 0.18, *p* = 0.05). Confidence was not significantly associated with gender, professional role, or length of employment.

**Conclusions:**

This study highlights the importance of job satisfaction and addiction-related training in strengthening community health workers’ confidence to deliver addiction services. Supportive work environments and strategies may be particularly critical for building a confident and effective community health workforce amid Vietnam’s recent health system reforms.

**Trial registration:**

Not applicable.

**Supplementary Information:**

The online version contains supplementary material available at https://doi.org/10.1186/s12913-026-15401-5.

## Introduction

Globally, substance use and related disorders remain major public health challenges, contributing to substantial burdens of morbidity, mortality, and social costs [1]. In this context, a growing body of evidence highlights that community health workers (CHWs) can play a vital role in expanding access to healthcare for people who use drugs (PWUDs) by providing health education, counseling, referral and linkage to specialized treatment, and adherence support, serving as a bridge between this underserved populations and the health systems to improve service uptake and engagement [2–4]. As CHWs often function as the first point of contact for basic health services at the community level, their confidence in delivering addiction-related care is critical, as greater confidence has been shown to increase providers’ willingness to take on expanded responsibilities and to deliver higher-quality services [5, 6]. Previous literature suggests that provider confidence is an important component of readiness to address substance use disorders and may contribute to improved service delivery and access to care [7, 8]. Evidence from Vietnam has also highlighted the importance of provider confidence in the delivery of MMT services, suggesting that strengthening confidence may support providers’ capacity to deliver addiction services in community-based settings [9]. Thus, identifying factors associated with CHWs’ confidence may inform workforce development and strengthening community-based addiction service delivery.

Prior research has also reported that provider-related factors such as training, professional attitudes, and job satisfaction are associated with confidence and readiness to implement new or expended health services [6, 9–12]. Research among nurses has demonstrated significant associations between self-efficacy and job satisfaction, as well as between communication confidence and professional performance, suggesting that supportive professional attitudes and workplace satisfaction contribute to higher provider confidence [10]. Training has also been shown to enhance provider competence and reduce stigmatizing attitudes. Some participatory approaches have increasingly been adapted from evidence-based HIV stigma reduction curricula to address drug use related stigma in healthcare settings [13, 14].

In Vietnam, drug use continues to represent a substantial public health challenge. As of February 2025, approximately 388,136 individuals were recorded as using illicit drugs, suspected of illicit drug use, or under post-rehabilitation supervision [15] with many at elevated risk for overdose, mental health concerns, and infectious diseases such as HIV. Although MMT and harm reduction programs have been scaled up nationally, limited empirical evidence exists on CHWs’ confidence in delivering addiction-related services and how this confidence is shaped by individual, attitudinal, and work-related factors. Most existing studies have focused on clinical providers or urban healthcare settings, leaving a gap in understanding care providers’ perspectives and experiences in community-level contexts. This knowledge gap is particularly salient considering Vietnam’s recent health system reforms. Effectively July 1, 2025, Vietnam implemented a major administrative restructuring, transitioning to a two-tier governance system comprising provincial and community levels [16]. Recently, Resolution No. 72-NQ/TW issued on September 9, 2025, reaffirmed the Government’s commitment to strengthening primary healthcare, with a particular emphasis on consolidating community-level health stations and enhancing the capacity of the grassroots health system to improve access and service delivery [17]. As these reforms are at an early stage of implementation, there remains limited evidence on how national policy directives are translated into practice at the community level, particularly for addiction-related services. Under this evolving system, the role and responsibilities of the community-level health services and CHWs are expected to expand. Drawing on previous studies of healthcare providers and implementation of addiction services, this study examined whether demographic characteristics, professional experience, previous addiction-related training, attitudes toward MMT, and job satisfaction were associated with CHWs’ confidence in addiction service provision. Study findings are expected to inform workforce development strategies and policy efforts to strengthen primary healthcare capacity for community-based addiction service delivery.

## Method

### Participants

This study analyzed baseline data from a randomized controlled trial conducted in three provinces of Vietnam: Ninh Binh, Da Nang, and Can Tho [18]. The parent trial was designed to evaluate the effects of the Community Care Consortium intervention; however, the present study is a cross-sectional analysis of baseline CHW data focused on identifying factors associated with confidence in addiction service provision rather than evaluating intervention effects. Data collection took place from May to July 2024 in 58 participating communities. To select communities, we first compiled a list of all communities in each province and ranked them in descending order based on the number of PWUDs. Communities with the largest PWUD populations were then selected for inclusion in the study. In the Vietnamese health system, community health centers (CHCs) typically staff between three and five CHWs, including doctors, assistant doctors, nurses, midwives, and pharmacists. Although MMT has been decentralized to selected CHCs in Vietnam, none of the participating CHCs in the present study directly provided MMT at the time of the assessment. Instead, participating CHWs were involved in broader addiction-related services, including counseling, referral, follow-up, and community-based support for PWUDs. Eligible CHWs were those aged 18 years or older who were actively involved in providing healthcare services within the community. Non-clinical personnel such as cleaners, accountants, and security staff were excluded. Project staff approached all eligible CHWs and explained the purpose and procedures of the study before obtaining written informed consent. A total of 175 eligible CHWs were approached, of whom 174 completed the baseline assessment, corresponding to a response rate of 99.4%. Ethical approval for all study procedures and instruments was obtained from the Institutional Review Boards (IRBs) of partner institutions in both Vietnam and the United States. Individuals who completed the assessment received a compensation of 230,000 VND (approximately USD $10).

### Data collection

During recruitment, project staff explained the purpose of the research, what participation involved, and any potential risks and benefits. Participation was voluntary, not linked to job duties, and CHWs were assured that their employment status would not be affected by their decision. Written informed consent was obtained prior to survey administration.

Data were collected using a CHW assessment survey developed specifically for this study. The assessments were conducted individually in private rooms at each community health center, and all study materials were administered in Vietnamese. Participants completed the survey using a Computer-Assisted Self-Interviewing (CASI) system, which displayed each question on a laptop screen and allowed participants to enter their responses directly. On average, participants required about 30 min to finish the survey. A trained interviewer was available nearby to assist with clarification of survey items or to address any technical issues with the CASI system if needed. A total of 174 CHWs participated in the study.

### Measures

Prior to the main study, all assessment measures were translated into Vietnamese using a forward- and back-translation process and pilot-tested with members of the target populations to ensure clarity, comprehension, cultural appropriateness, and relevance to the local context. Feedback from pilot participants was used to refine the wording and response options before the measures were administered in the main study.

*Confidence in providing addiction services* was assessed using a 13-item scale, developed by the study team and used in prior studies conducted in Vietnam [19, 20]. Participants indicated the extent to which they felt confident providing various addiction-related services, for example, “providing consultation and needed services to PWUDs,” “helping PWUDs adhere to the addiction treatment,” and “providing long-term follow-up and care for PWUDs.” Responses ranged from 1 = “Not confident at all” to 5 = “Very confident.” A total confidence score was generated by summing all 13 items, with higher scores indicating greater confidence (Cronbach’s α = 0.98).

*Job satisfaction* was evaluated using a four-item scale adapted from Karaferis et al. [21]. The items captured overall work experience and included statements such as: “You like doing the things you do at work,” “You feel a sense of pride in doing your job,” “Your job is enjoyable,” and “You feel that your job is meaningful.” Responses were provided on a five-point Likert scale ranging from 1 = “strongly agree” to 5 = “strongly disagree.” All items were reverse-coded, and a total score was calculated by summing the items, with higher scores reflecting higher levels of job satisfaction (Cronbach’s α = 0.93).

*Attitude towards MMT* was assessed using items adapted from work by Friedmann et al. [22] and Kruis et al. [23]. This eight-item scale measured CHWs’ perception to toward MMT. Example items included: “MMT reduces the negative consequences of using opioids,” “MMT reduces PWUDs’ HIV risk,” and “MMT is effective at reducing overdoses.” Participants rated each item on a 5-point Likert scale ranging from 1 = “strongly agree” to 5 = “strongly disagree.” All items were reverse-coded and summed, with higher total scores indicating a more positive attitude towards MMT (Cronbach’s alpha = 0.96). In addition, CHWs reported their demographic and professional backgrounds, including gender, age, job role, years of employment at the CHCs, and whether they had received training related to addiction services.

### Data analysis

Descriptive statistics were used to summarize demographic and occupational characteristics. Differences in mean confidence scores across categorical variables (gender, age group, occupation, years working at CHCs, and addiction training) were assessed using independent-sample *t* tests or one-way ANOVA, as appropriate. Post-hoc Bonferroni tests were applied following significant ANOVA results. For continuous correlates (job satisfaction and attitudes towards MMT), Pearson’s correlation coefficients (*r*) were estimated to examine their associations with confidence. To examine factors associated with CHWs’ confidence in providing addiction services, a series of multiple regression analyses was conducted. Model 1 included demographic and professional characteristics (gender, age, occupation, years of employment at CHCs, and addiction-related training). Attitudes toward MMT were added in Model 2, and job satisfaction was included in Model 3 to assess the robustness of these associations. Confidence score served as the dependent variable in all models. Results are reported as regression coefficients (β), standard errors (SE), and p-values. All analyses were conducted using Stata version 16.0.

## Results

As shown in Table 1, most participants were female (72.4%), with a mean age of 43.3 years (SD = 8.6). Approximately two-thirds of participants (66.7%) had more than 10 years of experience working at the CHCs, and about half of the respondents (46.6%) reported having received addiction-related training. Mean confidence score varied by age group (*p* < 0.01). CHWs aged 51 years or older reported higher level of confidence compared to those aged 18–34 years and 35–50 years. No significant differences were found by occupation or years of employment at CHCs. CHWs who had received addiction-related training reported significantly higher level of confidence compared to those without training (*p* < 0.01). Correlation analyses indicated modest but statistically significant positive associations between confidence in addiction service provision and both job satisfaction (*r* = 0.22, *p* < 0.01) and positive attitudes toward MMT (*r* = 0.21, *p* < 0.01).

**Table 1:** Participant characteristics and mean comparisons (*N* = 174)

	*N* / Mean (SD)	%	Confidence Score Scale Mean (SD)/ Pearson correlation (*r*)	*p*
Gender				
Male	48	27.6	45.7 (12.2)	0.14
Female	126	72.4	42.6 (12.1)	
Age	43.3 (8.6)			
18 to 34	33	19.0	41.2 (12.3)	< 0.01
35 to 50	95	54.6	41.9 (11.6)	
51 and above	46	26.4	48.4 (12.1)	
Occupation				
Doctor	36	20.7	42.8 (12.6)	0.9
Assistant doctor	67	38.5	43.8 (12.5)	
Nurse/Midwife	50	28.7	44.1 (12.3)	
Other	21	12.1	41.8 (10.2)	
Time in CHCs				
< 5 years	35	20.1	42.3 (10.8)	0.7
5–10 years	23	13.2	41.6 (15.0)	
11–20 years	67	38.5	43.7 (10.9)	
> 20 years	49	28.2	44.8 (13.4)	
Received addiction training				
Yes	81	46.6	46.1 (12.7)	< 0.01
No	93	53.4	41.2 (11.2)	
Job satisfaction	18.1 (2.1)		0.22	< 0.01
Attitude towards MMT	35.5 (5.1)		0.21	< 0.01

Mean confidence scores were compared using independent-samples t-tests for binary variables and one-way ANOVA for categorical variables with more than two categories. Pearson correlation coefficients (r) were calculated to assess associations between continuous variables and confidence scores

Results of multiple linear regression analyses are presented in Table 2. Across all models, older age was consistently associated with higher confidence scores. Receipt of addiction-related training also remained significantly associated with greater confidence. In Model 2, more positive attitudes toward MMT were associated with greater confidence (β = 0.18, *p* < 0.05); however, this relationship was attenuated and no longer statistically significant after job satisfaction was added in Model 3. Job satisfaction showed a positive association with confidence in Model 3 (β = 0.18, *p* = 0.05). Gender, professional role, and years of employment at the CHCs were not significantly associated with confidence in any model.

**Table 2:** Result from multiple regressions on CHWs’ confidence in providing addiction services

Characteristics	Model 1		Model 2		Model 3	
	β (SE)	*p*	β (SE)	*p*	β (SE)	*p*
Female	-0.07 (2.2)	0.4	-0.07 (2.1)	0.4	-0.10 (2.2)	0.2
Age	0.31 (0.2)	< 0.01	0.31 (0.2)	< 0.01	0.29 (0.2)	< 0.05
Doctor/assistant doctor *(vs. Nurse/Midwife/other)*	-0.09 (1.9)	0.2	-0.08 (1.9)	0.3	-0.08 (1.9)	0.3
Years at CHCs	-0.16 (0.1)	0.2	-0.18 (0.1)	0.1	-0.18 (0.1)	0.1
Received addiction training	0.18 (2.0)	< 0.05	0.16 (1.9)	< 0.05	0.16 (1.9)	< 0.05
Attitude towards MMT			0.18 (0.2)	< 0.05	0.07 (0.2)	0.4
Job satisfaction					0.18 (0.5)	0.05

β: Standardized coefficient; SE: Standard error

## Discussion

This study identified addiction-related training as a key factor associated with CHWs’ confidence in providing addiction services. This finding aligns with a body of evidence demonstrating that structured training interventions for healthcare providers and CHWs significantly improve their confidence, knowledge, and attitudes related to substance use disorder care. Prior studies in Vietnam and other settings have shown that training, particularly when it emphasizes communication skills, stigma reduction, and practical service delivery, can enhance providers’ interactions with PWUDs and strengthen perceived service delivery capacity [24–27]. However, nearly half of the CHWs in this study had not received addiction-related training, underscoring a substantial and actionable gap in workforce development at the community healthcare level.

This study also demonstrates that attitudes toward MMT play a role in shaping CHWs’ confidence, suggesting that favorable perception of MMT contribute to higher confidence in providing addiction services. This finding is consistent with prior studies showing that providers’ attitudes toward addiction treatment are closely linked to their confidence, readiness, and quality of service delivery [28–30]. More broadly, studies of addiction-related preparedness indicate interrelationships between attitudes and self-efficacy, reinforcing the importance of address stigma and beliefs [31]. These results support continued relevance of attitudinal intervention in the context of decentralized models of addiction care where CHWs play a frontline role.

Importantly, job satisfaction emerged as a significant correlated of CHWs’ confidence in providing addiction services and appeared to partially account for the association between attitudes toward MMT and confidence in the fully adjusted model. This finding may suggest that the influence of individual attitudes on confidence may be contingent on broader workplace conditions. Evidence from healthcare and other professional sectors consistently indicates that job satisfaction, organizational support, and perceived work environment quality are strongly linked to self-efficacy, engagement, and professional confidence [32–34]. Studies among nurses have similarly shown that higher job satisfaction is associated with stronger professional competence and occupational self-efficacy, particularly among more experienced staff [35, 36]. Together, these findings suggest that healthcare workers who experience higher levels of job satisfaction may be more motivated and engaged in delivering healthcare services, which in turn may enhance their confidence in service provision. The present findings extend this literature to CHWs delivering addiction services and suggest that confidence is shaped not only by individual knowledge or attitudes, but also by the organizational context in which care is delivered.

From an implementation perspective, these findings have important implications for Vietnam’s ongoing health system reforms, which increasingly emphasize community-level service delivery. As the responsibilities of community health centers expand, strategies to strengthen CHWs’ confidence must move beyond stand-alone training programs to include organizational interventions that enhance job satisfaction, including fair compensation, recognition of CHWs’ contributions, supportive supervision, and manageable workloads. Addressing both individual- and system-level determinates of confidence is important for sustaining high-quality addiction services at the community level.

This study has several limitations. First, the cross-sectional design limits the ability to infer causal relationships between training, job satisfaction, attitudes, and confidence in delivering addiction services. All measures were self-reported and may be subject to social desirability and recall bias. Second, although previous addiction-related training was associated with greater confidence, the study assessed only whether participants had ever received such training and did not collect detailed information regarding its content, duration, format, or recency. Therefore, we were unable to determine which specific types or components of training may be most relevant to CHWs’ confidence in addiction service provision. Third, although job satisfaction was identified as an important factor, this study did not collect detailed information on work environment characteristics that may influence CHWs’ job satisfaction. Factors such as workload, work-related stress, work pressure, organizational support, and other clinic-level characteristics were not assessed. Finally, the study did not assess participants’ years of experience in addiction-related service provision or the age at which they began working at community health centers. Therefore, although older age was associated with greater confidence, it was not possible to determine whether this association reflects greater professional experience, addiction-specific experience, or other age-related factors. Despite these limitations, the study contributes to the literature by highlighting the dual importance of addiction-related training and job satisfaction in shaping CHWs’ confidence to deliver addiction services. Future research should incorporate multilevel measures of organizational context to better understand and inform workforce strengthening strategies for addiction care in Vietnam.

## Conclusion

This study identifies addiction-related training and job satisfaction as key factors associated with CHWs’ confidence in delivering addiction services in Vietnam. Provider attitudes toward MMT also appear relevant, particularly within supportive work environment. These findings suggest that strengthening CHWs’ confidence requires not only technical training but also organizational strategies that support motivation and promote job satisfaction. Investing in both areas may enhance the effectiveness and sustainability of community-based addiction service delivery in Vietnam and similar low- and middle-income settings.

## Supplementary Information

Below is the link to the electronic supplementary material.

Supplementary Material 1

## Data Availability

Data are available upon reasonable request after the completion of the trial.

## Abbreviations

CHWs: Community health workers
PWUDs: People who use drugs
MMT: Methadone maintenance treatment
CHCs: Community health centers

## References

[1] Dongying C, Yanyan S, Xiaowu L, Zongyi Y. Global burden on drug use disorders from 1990 to 2021 and projections to 2046. Front Public Health. 2025;13:1550518. 10.3389/fpubh.2025.1550518 PMC12336172 40791629

[2] Morgan K, Lee J, Sebar B. Community health workers: a bridge to healthcare for people who inject drugs. Int J Drug Policy. 2015;26(4):380–7. 10.1016/j.drugpo.2014.11.001 25477284

[3] Hirschhorn LR, Govender I, Zulu JM. Community health workers: essential in ensuring primary health care for equitable universal health coverage, but more knowledge and action is needed. BMC Prim Care. 2023;24(1):219. 10.1186/s12875-023-02175-6 PMC10599034 37880575

[4] Carson SL, Hong C, Behforouz H, Chang E, Dixon LZ, Factor D, et al. Mechanisms for Community Health Worker Action on Patient-, Institutional-, and Community-Level Barriers to Primary Care in a Safety-Net Setting. J Ambul Care Manage. 2022;45(1):22–35. 10.1097/JAC.0000000000000405 PMC8622376 34812754

[5] Baek OJ, Shin SH. Factors Influencing Nursing Students’ Willingness to Participate in Patient Safety: The Role of Patient Safety Confidence and Campaign Participation. SAGE Open Nurs. 2025;11:23779608251321359. 10.1177/23779608251321359 PMC11826861 39958189

[6] Kim MK, Arsenault C, Atuyambe LM, Macwan’gi M, Kruk ME. Determinants of healthcare providers’ confidence in their clinical skills to deliver quality obstetric and newborn care in Uganda and Zambia. BMC Health Serv Res. 2020;20(1):539. 10.1186/s12913-020-05410-3 PMC7296707 32539737

[7] Turi ER, McMenamin A, Wolk CB, Poghosyan L. Primary care provider confidence in addressing opioid use disorder: A concept analysis. Res Nurs Health. 2023;46(2):263–73. 10.1002/nur.22294 PMC10033432 36611290

[8] Brady BR, Murphy BS, Padilla AR, Clichee DM, Cameron E, Mendivil E, et al. Collaborative consultation mentorship increases provider confidence to prescribe medications for opioid use disorder. J Opioid Manag. 2025;21(6):471–6. 10.5055/jom.0976 41506693

[9] Nguyen BD, Li L, Lin C, Nguyen TT, Shoptaw S, Le MG. Confidence in providing methadone maintenance treatment of primary care providers in Vietnam. Addict Sci Clin Pract. 2024;19(1):43. 10.1186/s13722-023-00419-5 PMC11112890 38778428

[10] Dautovic A, Fredriksson-Larsson U, Yang Hansen K, Brink E. Occupational self-efficacy, job satisfaction and learning potential of the workplace in a sample of diabetes specialist nurses: A structural equation modeling analysis. Int J Nurs Stud Adv. 2025;9:100446. 10.1016/j.ijnsa.2025.100446 PMC12639621 41281386

[11] Ghahremani L, Khoramaki Z, Kaveh MH, Karimi M, Nazari M, Orgambídez Ramos A. Communication Self-Efficacy and Job Satisfaction among Nurses during the COVID-19 Pandemic. J Nurs Manag. 2024;2024:8869949. 10.1155/2024/8869949 PMC11918967 40224843

[12] Chang MC, Hsieh JG, Wei MH, Tsai CH, Yu JH, Wang YW. Familiarity, attitude, and confidence of health literacy practice among community healthcare providers in Taiwan. Int J Environ Res Public Health. 2021;18(23). 10.3390/ijerph182312610 PMC8656928 34886334

[13] Madden EF, Qeadan F, Frabis F, Cohn J, Barbeau W, Greenwald MK. Reducing stigma in primary care toward people who use drugs: A pilot study exploring the promise and limits of stigma training. J Subst Use Addict Treat. 2025;176:209748. 10.1016/j.josat.2025.209748 PMC12954461 40581186

[14] Mlunde LB, Saalim K, Mbwambo JK, Kiwia P, Fitch E, Manyama W, et al. Adapting a health facility HIV stigma-reduction participatory training intervention to address drug use stigma in HIV care and treatment clinics in Dar es Salaam, Tanzania. Harm Reduct J. 2024;21(1):65. 10.1186/s12954-024-00965-4 PMC10941424 38491349

[15] Trang NT, Diep NB, Thai PK, Duc NC, Giang LM. Perspectives on addiction-related problems in Vietnam. Addiction. 2026;121(2):448–57. 10.1111/add.70207 PMC12779594 41105039

[16] News LD. International press conference announces the operation of Vietnam’s new organizational apparatus from 1.7.2025 [Internet]. 2025 [cited 2026 Aug 14]. Available from: https://news.laodong.vn/thoi-su/hop-bao-quoc-te-cong-bo-van-hanh-to-chuc-bo-may-moi-cua-viet-nam-tu-172025-1525213.ldo.

[17] S News. New resolution puts people’s health at the forefront [Internet]. 2025 [cited 2026 Aug 14]. Available from: https://en.sggp.org.vn/resolution-no-72-places-peoples-health-at-the-centre-of-development-strategy-post120282.html.

[18] Li L, Nguyen TA, Liang LJ, Lin C, Pham TH, Nguyen HTT, et al. Strengthening Addiction Care Continuum Through Community Consortium in Vietnam: Protocol for a Cluster-Randomized Controlled Trial. JMIR Res Protoc. 2023;12:e44219. 10.2196/44219 PMC10131887 36947125

[19] Li L, Lin C, Liang LJ, Chen J, Feng N, Nguyen AT. HIV- and Drug Use-Related Stigma and Service Provision Among Community Health Workers in Vietnam. AIDS Educ Prev. 2020;32(2):169–79. 10.1521/aeap.2020.32.2.169 PMC8211404 32539482

[20] Li L, Lin C, Pham LQ, Nguyen DB, Le TA. Networking community health workers for service integration: role of social media. AIDS Care. 2023;35(7):1030–6. 10.1080/09540121.2021.2019668 PMC9271530 35014579

[21] Karaferis D, Aletras V, Niakas D. Determining dimensions of job satisfaction in healthcare using factor analysis. BMC Psychol. 2022;10(1):240. 10.1186/s40359-022-00941-2 PMC9610349 36303222

[22] Friedmann PD, Wilson D, Knudsen HK, Ducharme LJ, Welsh WN, Frisman L, et al. Effect of an organizational linkage intervention on staff perceptions of medication-assisted treatment and referral intentions in community corrections. J Subst Abuse Treat. 2015;50:50–8. 10.1016/j.jsat.2014.10.001 PMC4304936 25456091

[23] Kruis NE, McLean K, Perry P. Exploring first responders’ perceptions of medication for addiction treatment: Does stigma influence attitudes? J Subst Abuse Treat. 2021;131:108485. 10.1016/j.jsat.2021.108485 34098289

[24] Li L, Hien NT, Liang LJ, Lin C, Lan CW, Lee SJ, et al. Efficacy of Communication Training of Community Health Workers on Service Delivery to People Who Inject Drugs in Vietnam: A Clustered Randomized Trial. Am J Public Health. 2018;108(6):791–8. 10.2105/AJPH.2018.304350 PMC5944870 29672144

[25] Xia W, Li HCW, Liang T, Luo Y, Ho LLK, Cheung AT, et al. Structured online training for university students to deliver peer-led addiction counselling for young drug abusers in China: Effect on improving knowledge, attitude, confidence, and skills. Patient Educ Couns. 2022;105(4):1009–17. 10.1016/j.pec.2021.07.038 34334262

[26] Li L, Lin C, Liang LJ, Nguyen DB, Pham LQ, Le TA, et al. Community Capacity Building for HIV and Addiction Service Integration: An Intervention Trial in Vietnam. AIDS Behav. 2022;26(1):123–31. 10.1007/s10461-021-03363-0 PMC8733056 34228251

[27] Zoorob R, Kim-Vences S, Hirth JM, Schaper K, Grigoryan L, Gonzalez S, et al. Asynchronous online training increased knowledge, confidence, and intent to screen and treat substance use disorders among primary care clinicians practicing in Texas. Prev Med. 2024;185:108038. 10.1016/j.ypmed.2024.108038 PMC12145182 38857769

[28] Pinxten WJL, Fitriana E, Jokūbonis D, Adomaitiene V, Leskauskas D, Hutschemaekers GJM, et al. Physicians’ attitudes towards patients with substance-related disorders predict training needs in addiction medicine: challenges and opportunities for strengthening the global addiction medicine workforce. BMC Med Educ. 2025;25(1):896. 10.1186/s12909-024-05109-7 PMC12211938 40598317

[29] Wakeman SE, Pham-Kanter G, Donelan K. Attitudes, practices, and preparedness to care for patients with substance use disorder: Results from a survey of general internists. Subst Abus. 2016;37(4):635–41. 10.1080/08897077.2016.1187240 27164025

[30] Machavariani E, Bromberg DJ, Dumchev K, Esserman D, Earnshaw VA, Pykalo I, et al. Decrease in provider stigma is associated with improved quality health indicators among individuals receiving methadone in primary care centers in Ukraine. Int J Drug Policy. 2025;136:104682. 10.1016/j.drugpo.2024.104682 PMC11821433 39705875

[31] Fooladvand K. Investigating the Role of Attitude towards Substance use in the Relationship between Students’ Self-Efficacy and Preparedness for Addiction. Int J Biol Sci. 2020;13:129–34.

[32] Al-Hamdan Z, Bani Issa H. The role of organizational support and self-efficacy on work engagement among registered nurses in Jordan: A descriptive study. J Nurs Manag. 2022;30(7):2154–64. 10.1111/jonm.13456 34415087

[33] Karaferis D, Aletras V, Raikou M, Niakas D. Factors Influencing Motivation and Work Engagement of Healthcare Professionals. Mater Sociomed. 2022;34(3):216–24. 10.5455/msm.2022.34.216-224 PMC9559882 36310751

[34] Zhang J, He W, Jiang J, Luo X, Li G, Li Y, et al. The Impact of high-performance work system perceived by medical staff on job satisfaction: the mediating role of self-efficacy. Psychol Health Med. 2024;29(3):492–504. 10.1080/13548506.2023.2189271 36916209

[35] Kallerhult Hermansson S, Norström F, Hilli Y, Rennemo Vaag J, Bölenius K. Job satisfaction, professional competence, and self-efficacy: a multicenter cross-sectional study among registered nurses in Sweden and Norway. BMC Health Serv Res. 2024;24(1):734. 10.1186/s12913-024-11177-8 PMC11179280 38877558

[36] Ghaleh RJ, Mohsenipouya H, Hosseinnataj A, Al-Mamun F, Mamun MA. Job satisfaction and the role of self-esteem and self-efficacy: A cross-sectional study among Iranian nurses. Nurs Open. 2024;11(6):e2215. 10.1002/nop2.2215 PMC11180991 38881516

