# Supplementary material for: Factors associated with community health workers’ confidence in addiction service provision in Vietnam: a cross-sectional study
Source: BMC Health Serv Res. 2026 Aug 18;26:1596. doi: 10.1186/s12913-026-15401-5 (PMC13761144; doi:10.1186/s12913-026-15401-5)
Supplement: Supplementary file 1 [file 12913_2026_15401_MOESM1_ESM.docx]

**CHW Questionnaire**

Province________________

CHC code: ______________	Participant ID: ______________________

Interview date: __/__/____(dd/mm/yyyy)

**A. Demographics**

**To start, we would like to ask some questions about yourself.**

1. What is your gender?	1. Male	2. Female
2. What year were you born? |___|___|___|___| year
3. What is your ethnicity?

1. Kinh

2. Mường

3. Khmer

4. Chăm

5. SánDìu

6. Sán Chay

7. Tày

8. Nùng

9. Dao

10. Ê đê

6. Other, please specify

4. How many years of schooling have you completed? (from primary school)________years
5. What is your highest medical training?

1.	Graduate (4-6 years in medicine or pharmacy) and above

2.	College (3 years in medicine or pharmacy)

3.	Intermediate (2 years in medicine or pharmacy)

4.	Primary (vocational school, with certificate: 6 months – 1 year)

5.	Short-term training (no certificate: less than 6 months)

6. Which one of the following categories do you belong to?

1.	Doctor

2.	Assistant doctor

3.	Nurse

4.	Midwife

5.	Pharmacist

6.	Other, please specify_______

7. How long have you worked in the medical field?	____ years	____ months
8. How long have you been working at this Commune Health Center (CHC)? ____years ___months
9. Have you received any addiction treatment-related training since working at this CHC?
   1. Yes	2. No

9a. If "Yes", how many times? _________

10. Have you received any HIV/AIDS-related training since working at this CHC?
1. Yes	2. No

10a. If "Yes", how many times? _________

**B. Health services**

**Please fill in your answers regarding the services you provided for your patients in the past 3 months.**

1.	How many patients in total did you provide health services to in the past 3 months? _____patients

2. How many patients with HIV did you provide health services to in the past 3 months? ______patients
3. How many patients who use drugs did you provide health services to in the past 3 months? ______patients
4. How many patients living with HIV who use drugs did you provide health services to in the past 3 months? _____patients
5. Did you provide the following services to your patients in the past 3 months? If yes, how many times?

| Services | Yes | How many times | No |  |
| --- | --- | --- | --- | --- |
| a Community outreach that is related to substance use and/or HIV |  | ______times |  |  |
| b HIV testing |  | ______times |  |  |
| c CD4/viral load testing |  |  |  |  |
| d Making referral to ART outpatient clinics (OPC) |  | ______times |  |  |
| e Distributing ART medication |  | ______times |  |  |
| f Conducting urine test for substance use |  | ______times |  |  |
| g Providing methadone maintenance therapy (MMT) and/or needle and syringe provision (NSP) |  | ______times |  |  |
| h Making referrals to MMT, or other addiction treatment |  | ______times |  |  |
| i Testing and screening of other infectious diseases (such as STI, HBV, HCV, and TB) |  | ______times |  |  |
| j Treating infectious diseases |  | ______times |  |  |
| k Making referrals to infectious disease hospitals |  | ______times |  |  |
| l Screening for mental illness |  | ______times |  |  |
| m Providing psychological counseling |  | ______times |  |  |
| n Making referral to mental health services |  | ______times |  |  |
| o Conducting home visits for patients who use drugs |  | ______times |  |  |
| p Providing counseling/reminders to support patients who use drug’s treatment adherence |  | ______times |  |  |
| q Providing other health services for patients who use drugs, such as basic preventive/therapeutic service |  | ______times |  |  |

6. In the past 3 months, have you used the following channels to communicate with patients who use drugs (PWUD)? If yes, how many times?

| Channels | Yes | How many times | No |
| --- | --- | --- | --- |
| a Having one-on-one meeting |  | ______times |  |
| b Conducting group education activities |  | ______times |  |
| c Via phone call |  | ______times |  |
| d Via text message |  | ______times |  |
| e Via online platform/smartphone apps |  | _____times |  |
| f Home visits |  | _____times |  |
| g Community outreach activities |  | _____times |  |

7. Do you consider the following barriers to providing MMT or other addiction services in community settings?

a Little or no perceived need	Yes	No
b Unavailability of a physician willing/able to administer it	Yes	No
c Lack of staff	Yes	No
d Lack of funding	Yes	No
e Staff resistance	Yes	No
f Patient resistance	Yes	No
g Community resistance	Yes	No
h Other, Specify ___________

**C. Job Satisfaction/Attitude/Belief/Stigma**

1. Please tell us how much you agree or disagree with the following statements about your job.

|  |  | Strongly agree | Agree | Not sure | Disagree | Strongly disagree |
| --- | --- | --- | --- | --- | --- | --- |
| a You feel that your job is meaningful. | | 1 | 2 | 3 | 4 | 5 |
| b You like doing the things you do at work. | | 1 | 2 | 3 | 4 | 5 |
| c You feel a sense of pride in doing your job. | | 1 | 2 | 3 | 4 | 5 |
| d Your job is enjoyable. | | 1 | 2 | 3 | 4 | 5 |

2. Please tell us how much you agree or disagree with the following statements.

|  | Strongly agree | Agree | | Not sure | | Disagree | | Strongly disagree |
| --- | --- | --- | --- | --- | --- | --- | --- | --- |
| a MMT reduces the negative consequences of using opioids. | 1 | | 2 | | 3 | | 4 | 5 |
| b MMT reduces PWUD's criminal activities. | 1 | | 2 | | 3 | | 4 | 5 |
| c MMT reduces PWUD's HIV risk. | 1 | | 2 | | 3 | | 4 | 5 |
| d MMT reduces PWUD's risk of dying. | 1 | | 2 | | 3 | | 4 | 5 |
| e MMT reduces PWUD's consumption of illicit opioids. | 1 | | 2 | | 3 | | 4 | 5 |
| f MMT is effective at reducing overdoses. | 1 | | 2 | | 3 | | 4 | 5 |
| g MMT is effective at reducing future crime. | 1 | | 2 | | 3 | | 4 | 5 |
| h MMT is a good investment for society. | 1 | | 2 | | 3 | | 4 | 5 |

3. Please tell us your opinion about harm reduction concept related to drug use and addiction.

|  | | Strongly agree | | | Agree | | | Not sure | | | Disagree | | | Strongly disagree | | |
| --- | --- | --- | --- | --- | --- | --- | --- | --- | --- | --- | --- | --- | --- | --- | --- | --- |
| a Substance abuse is a chronic disease. | 1 | | | 2 | | | 3 | | | 4 | | | 5 | | |  |
| b Teaching drug users to inject safely is a good social work practice. | 1 | | | 2 | | | 3 | | | 4 | | | 5 | | |  |
| c Relapsing individuals should be allowed to remain in MMT for substance abuse. | 1 | | | 2 | | | 3 | | | 4 | | | 5 | | |  |
| d All individuals who want to enroll in MMT should receive it. | 1 | | | 2 | | | 3 | | | 4 | | | 5 | | |  |
| e Reducing drug use's harmful consequences is as important as achieving abstinence. | 1 | | | 2 | | | 3 | | | 4 | | | 5 | | |  |
| f PWUD should be praised for making changes such as cutting down on their drug consumption or switching from injectable drugs to oral drugs. | 1 | | | 2 | | | 3 | | | 4 | | | 5 | | |  |
| g MMT should be available as a lifelong treatment option. | 1 | | | 2 | | | 3 | | | 4 | | | 5 | | |  |
| h Needle and syringe provision encourage illicit drug use. | 1 | | | 2 | | | 3 | | | 4 | | | 5 | | |  |
| i The primary goal of MMT should be abstaining from all substances. | 1 | | 2 | | | 3 | | | 4 | | | 5 | | |  |  |
| j Harm reduction is synonymous with legalization of drugs. | 1 | | 2 | | | 3 | | | 4 | | | 5 | | |  |  |

4. Please tell us about your perception toward people who use drugs in general.

|  | Not at all | A little bit | Moderately | Quite a bit | Very much |
| --- | --- | --- | --- | --- | --- |
| a To what extent is an individual personally responsible for his/her problematic drug use? | 1 | 2 | 3 | 4 | 5 |
| b To what extent do you feel angry towards people who use drugs? | 1 | 2 | 3 | 4 | 5 |
| c To what extent do you feel disappointed towards people who use drugs? | 1 | 2 | 3 | 4 | 5 |
| d To what extent do you feel sympathetic towards people who use drugs? | 1 | 2 | 3 | 4 | 5 |
| e To what extent do you feel concerned about people who use drugs? | 1 | 2 | 3 | 4 | 5 |
| f To what extent do people who use drugs deserve the same level of medical care as people who do not use drugs? | 1 | 2 | 3 | 4 | 5 |

**D. Confidence/Support**

1. Please indicate how confident you are in providing the following services to PWUD and family members.

|  | Not confident at all | | A Little bit confident | | Moderately confident | | Quite a bit confident | | Very confident | |  |
| --- | --- | --- | --- | --- | --- | --- | --- | --- | --- | --- | --- |
| a Getting in touch with PWUD in the community in the community | 1 | | 2 | | 3 | | 4 | | 5 | | |
| b Providing consultation and needed services to PWUD | 1 | | 2 | | 3 | | 4 | | 5 | | |
| c Evaluating PWUD’s comprehensive service needs | 1 | | 2 | | 3 | | 4 | | 5 | | |
| d Tailoring treatment and care based on PWUD’s needs | 1 | | 2 | | 3 | | 4 | | 5 | | |
| e Engaging PWUD to addiction treatment and care | 1 | | 2 | | 3 | | 4 | | 5 | | |
| f Helping PWUD adhere to the addiction treatment | 1 | | 2 | | 3 | | 4 | | 5 | | |
| g Motivating PWUD to make positive behavior change | 1 | | 2 | | 3 | | 4 | | 5 | | |
| h Providing long-term follow-up and care for PWUD | 1 | | 2 | | 3 | | 4 | | 5 | | |
| i Supporting PWUD and family members to tackle stigma and discrimination | 1 | | 2 | | 3 | | 4 | | 5 | | |
| j Helping to address problems faced by PWUD and family members | 1 | | 2 | | 3 | | 4 | | 5 | | |
| k Visiting PWUD and family members in their home setting | 1 | | 2 | | 3 | | 4 | | 5 | | |
| l Motivating PWUD and family member to participate in community activities | 1 | 2 | | 3 | | 4 | | 5 | |  |  |
| m Engaging family members to support PWUD’s health service utilization | 1 | 2 | | 3 | | 4 | | 5 | |  |  |

2. Please tell us how often you have made following efforts for your PWUD patients in the past 3 months.

|  | None of the time | A little of the time | Some of the time | Most of the time | All the time |
| --- | --- | --- | --- | --- | --- |
| a How often did you provide counseling to PWUD patients? | 1 | 2 | 3 | 4 | 5 |
| b How often did you answer PWUD patients' questions? | 1 | 2 | 3 | 4 | 5 |
| c How often did you motivate PWUD to seek addiction treatment? | 1 | 2 | 3 | 4 | 5 |
| d How often did you encourage PWUD patients to stay in addiction treatment? | 1 | 2 | 3 | 4 | 5 |
| e How often did you ask about the reason if PWUD patients missed an appointment? | 1 | 2 | 3 | 4 | 5 |
| f How often did you remind PWUD patients to have regular tests for prevention and treatment? | 1 | 2 | 3 | 4 | 5 |
| g How often did you talk with PWUD about the harm of untreated addiction/drug use? | 1 | 2 | 3 | 4 | 5 |
| h How often did you praise PWUD when he/she make progress on seek or stay in treatment? | 1 | 2 | 3 | 4 | 5 |
| i How often did you help PWUD/family members to deal with stress and burden in family? | 1 | 2 | 3 | 4 | 5 |
| j How often did you visit PWUD patient's home and check with the family situation? | 1 | 2 | 3 | 4 | 5 |

**We have a few more questions regarding your evaluation of this questionnaire.**

1. Do you think that the questions are clear and easily understood?

1.   Yes	2.     Not sure	3.     No

2. Do you think people will answer them in an honest manner?

1.    Yes	2.     Not sure	3.     No

3. Is there a particular section or question you have trouble understanding or answering?

Questions: ___________

Comments: ________________

**Thank you very much for your time. Your answers are very helpful!**

**[For Data Collection Team]**

Total time completing the questionnaire	______ min

Any questions raised by the participant ______________________________________

Any technical issues _____________________________________________

Other comments/observations______________________________________
